# SIR epidemics and vaccination on random graphs with clustering

**DOI:** 10.1007/s00285-019-01347-2

**Published:** 2019-04-10

**Authors:** Carolina Fransson, Pieter Trapman

**Affiliations:** Department of Mathematics, Stockholm University, 106 91 Stockholm, Sweden

**Keywords:** SIR epidemics, Configuration model, Clustering, Branching processes, Vaccination

## Abstract

In this paper we consider Susceptible $\rightarrow$ Infectious $\rightarrow$ Recovered (SIR) epidemics on random graphs with clustering. To incorporate group structure of the underlying social network, we use a generalized version of the configuration model in which each node is a member of a specified number of triangles. SIR epidemics on this type of graph have earlier been investigated under the assumption of homogeneous infectivity and also under the assumption of Poisson transmission and recovery rates. We extend known results from literature by relaxing the assumption of homogeneous infectivity both in individual infectivity and between different kinds of neighbours. An important special case of the epidemic model analysed in this paper is epidemics in continuous time with arbitrary infectious period distribution. We use branching process approximations of the spread of the disease to provide expressions for the basic reproduction number $R_0$, the probability of a major outbreak and the expected final size. In addition, the impact of random vaccination with a perfect vaccine on the final outcome of the epidemic is investigated. We find that, for this particular model, $R_0$ equals the perfect vaccine-associated reproduction number. Generalizations to groups larger than three are discussed briefly.

## Introduction

One of the most important factors that determine the fate of an outbreak of an infectious disease is the contact pattern of individuals in the population. The frequency and duration of the contacts between individuals typically depend on the nature of their relationship. For this reason, recent interest has focused on the impact of the underlying social network on the spread of the disease. The social network is typically represented by a random graph (Newman et al. 2002), in which the nodes or vertices represent individuals and the edges represent social contacts between the individuals. Two nodes that share an edge are called “neighbours”.

A popular choice when generating random graphs with a specified degree distribution is the configuration model (CM). It was introduced by Bollobás (1980) for the special case where the degree distribution is degenerate (i.e. every node of the graph has the same degree) and extended to more general degree distributions by Molloy and Reed (1995, 1998). There is a vast literature on epidemics on configuration model graphs [see e.g. Andersson (1999), Britton et al. (2007), Janson et al. (2014), Barbour and Reinert (2013), Bhamidi et al. (2014)].

An important feature of the configuration model is that, under mild regularity conditions on the degrees, this type of graph is asymptotically unclustered. That is to say, it contains virtually no groups and short circuits. Real world networks do, however, typically exhibit clustering (Newman 2003), and there are a number of graph models that do allow for group structure (Bollobás et al. 2011; Karoński et al. 1999; Newman 2002). Epidemics on graphs with group structure were studied by Trapman (2007); Ball et al. (2009, 2010, 2014); Coupechoux and Lelarge (2015); Britton et al. (2008).

In this paper, we use a generalized version of the configuration model to incorporate clustering of the social network in the analysis of the spread of an infectious disease. The configuration model with clustering (CMC) was independently introduced by Miller (2009) and Newman (2009). It is an extension of the CM in the sense that, for each node *u*, in addition to the degree of *u* one also specifies the number of pairs of neighbours of *u* that are in turn neighbours of each others. In other words, one specifies the number of triangles (with non-overlapping edges) of which *u* is a member [see Sect. 2.1 for a precise definition of the graph model]. This allows for graphs with non-negligible clustering and a specified degree distribution. That is to say, the CMC deviates from the classical Erdős–Rényi graph model (Erdős and Rényi 1959) in two fundamental ways: it allows for for a non-Poissonian degree distributions and is asymptotically clustered. Epidemics on this type of graph have previously been studied by Miller (2009) and Volz (2011). Miller (2009) investigated the impact of clustering on the epidemic threshold, formulated as a bond percolation problem. This means that the infectivity of infected individuals is assumed to be homogeneous; an infected individual transmits the disease to each of its neighbours independently with some fixed probability *T*. Volz (2011) investigated the time evolution and final size of epidemics on CMC graphs under the assumption of exponentially distributed infectious periods during which individuals contact neighbours at a constant rate.

The main contribution of our research is that we extend the results of Miller (2009) and Volz (2011) by allowing for heterogeneous infectivity, i.e. by allowing for some infected individuals to be more contagious than others or for individuals to exhibit different contact behaviors for different types of neighbours. Such heterogeneity may, for instance, reflect variability in the infectious period or contact preferences on the part of individuals. We provide expressions for the probability of a major outbreak and the final size of a major outbreak. A key tool in our analysis is the approximation of the epidemic seen from a “generation of infection” or “rank” perspective by a multitype Galton Watson branching process. This approximation, which is interesting in its own right, gives rise to the rank-based reproduction number $R_0$ [see e.g. Pellis et al. (2008, 2012)]. We note that especially allowing for heterogeneity in the infectivity of individuals requires a more intricate branching process approximation than a model with homogeneous infectivity [as analysed by Miller (2009)]. To see this, consider an individual *v* which is infected through a triangle $\varDelta$. The “local epidemics” in $\varDelta$ and in other triangles *v* is part of all depend on the infectivity of *v* and are therefore in general not independent.

The second contribution of this paper concerns vaccination. We investigate the impact of uniform vaccination (i.e. vaccinated individuals are selected uniformly at random) with a perfect vaccine (i.e. a vaccine that provides full and permanent immunity to the disease). We find that it is necessary to vaccinate a fraction $1-1/R_0$ of the population in order to prevent a major outbreak of the disease, as in the case of homogeneous mixing. We illustrate our findings with numerical examples.

This paper is structured as follows. In Sect. 2 we provide the preliminaries for the model. In Sect. 2.1 we give a more detailed description of how graphs are generated in the CMC and investigate the asymptotic clustering of such graphs and in Sect. 2.2 the epidemic model is specified. Sections 2.3 and 2.4 contains an overview of the concept of reproduction numbers and the necessary branching process background. In Sect. 3, we derive expressions for the probability of a major outbreak and the expected final size under the assumption of an unvaccinated and fully susceptible population, and in Sect. 4 the analysis is repeated under the assumption of uniform vaccination with a perfect vaccine. We illustrate our findings with numerical examples presented in Sect. 5 and discuss possible extensions in Sect. 6.

## Preliminaries

### The configuration model with clustering

**Fig. 1:**
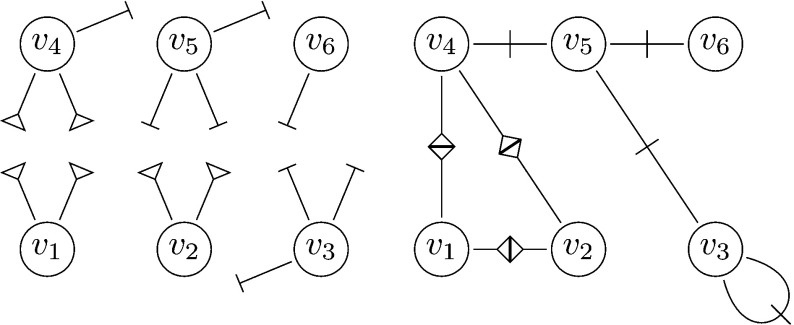
Schematic illustration of the construction of a CMC graph. Triangle half-edges (marked with a triangle) and single half-edges (marked with a perpendicular line) are assigned to the nodes of the graph (left). The half-edges are then matched uniformly at random (right). Note that two of the half-edges attached to $v_3$ are paired with each other and so form a self-loop

A CMC graph is constructed as follows. Let $\{p(k_s, k_{\varDelta })\}_{k_s,k_{\varDelta }\in \mathbb {N}_0}$ be a prescribed joint degree distribution, where $k_s$ denotes the number of single edges attached to a node, and $k_{\varDelta }$ denotes the number of pairs of triangle edges. Throughout, $(S,\varDelta )$ is assumed to be a generic random vector distributed according to *p*. Let $\{(S_i, \varDelta _i)\}_{i=1}^N$ be a sequence of independent copies of $(S,\varDelta )$. Analogously to the CM, a graph $G_N=G_N(p)$ of size *N* is constructed by first assigning the single degree $S_i$ and the triangle degree $\varDelta _i$ to the node $v_i$, $i=1,2,\ldots ,N$. One may think of this step in terms of half-edges; to each node $v_i$, we attach $S_i$ single half-edges and $\varDelta _i$ pairs of triangle half-edges. The single half-edges are then matched in pairs and the triangle half-edge pairs in threes by choosing a matching uniformly at random among all possible such matchings. The process of joining half-edges is illustrated in Fig. 1. As described in Miller (2009), the matching may be carried out as follows. Two lists of nodes, one single degree list and one triangle degree list are created. A node with joint degree $(k_s,k_{\varDelta })$ appears $k_s$ times in the single list and $k_{\varDelta }$ times in the triangle list. The lists are then shuffled uniformly, and the nodes on positions $2m+1$ and $2m+2$ in the single degree list and positions $3m+1,\ 3m+2$ and $3m+3$ in the triangle degree list are matched, $m\in \mathbb {N}_0$.

We define the *total single degree* as

$$\begin{aligned} D^{(N)}_S:=\sum _{i=1}^NS_i \end{aligned}$$

and the *total triangle degree* as

$$\begin{aligned} D^{(N)}_{\varDelta }:=\sum _{i=1}^N\varDelta _i. \end{aligned}$$

If the total single degree (that is, the length of the single degree list) is not even or if the total triangle edge degree (the length of the triangle degree list) is not a multiple of three we erase a single half-edge and/or one or two triangle half-edge pairs chosen uniformly at random. Similarly, we erase self-loops and merge multiple edges, so that the resulting graph is simple. Under assumption A1 (stated below) on *p* it holds that the number of single self-loops and single double edges converge in distribution to independent Poisson random variables with finite means [cf. Van der Hofstad (2016, prop. 7.13)].

For this reason, self-loops and multiple edges are negligible in the limit as $N\rightarrow \infty$. In the remainder of this paper, we ignore the small differences in the topology of the graph that arise from erasing multiple edges or self-loops. In addition, we ignore the small differences in effective degree distribution that arise from erasing half-edges so that the number of single and triangle half-edges are multiples of two and three, respectively.

We make the following assumptions on *p*.

1. $$E(\varDelta ^2)<\infty \text { and } E(S^2)<\infty . $$
2. $$P(\max (\varDelta ,S)\ge 2)>0$$ and $$E( \varDelta S)>0$$.

Note that the assumption A1 implies $E(\varDelta S)<\infty$. Assumption A2 ensures that the mean matrices of the approximating branching processes (presented below) are positively regular (we say that an $r\times r$ matrix *M* is positively regular if it has finite non-negative entries and for some $n\in \mathbb {N}$ all entries of $M^n$ are strictly positive).

#### Clustering coefficient of $G_N$

For any undirected graph we can measure the amount of clustering in the network using the so-called clustering coefficient, which is defined as follows. Let $G=(V, E)$ be an undirected graph with node set *V* and edge set *E*. Define

$$\begin{aligned} \mathcal {W}^G_{\wedge }=\{(u,v,w)\in V^3: (u,v), (v,w)\in E\} \end{aligned}$$

the set of all ordered wedges (i.e. directed paths consisting of precisely two edges) of *G* and

$$\begin{aligned} \mathcal {W}_{\varDelta }^G=\{(u,v,w)\in V^3: (u,v), (v,w), (w, u)\in E\}\subset \mathcal {W}_{\wedge }^G \end{aligned}$$

the set of all ordered triangles of *G*. The *clustering coefficientC*(*G*) of *G* is a measure of the degree of clustering of *G* and is defined as the fraction of the ordered wedges of *G* that are also triangles:

$$\begin{aligned} C(G)=\frac{\vert \mathcal {W}_{\varDelta }^G\vert }{\vert \mathcal {W}^G_{\wedge }\vert }. \end{aligned}$$

      Here $|\cdot |$ denotes the cardinality of a set.

As stated in the following proposition, CMC graphs have asymptotically non-zero clustering as $N\rightarrow \infty$. An analogous result for fixed degree sequences was presented in Newman (2009). Let $\overset{P}{\longrightarrow }$ denote convergence in probability.

##### Proposition 1

Let $\{G_N\}_N$ be a sequence of CMC graphs with independent degrees drawn from *p*. If *p* satisfies assumption A1 then

1 $$\begin{aligned} C(G_N)\overset{P}{\longrightarrow } \frac{E(2\varDelta )}{E((2\varDelta +S)^2)-E(2\varDelta +S)}. \end{aligned}$$

The proof is presented in the Appendix.

#### Downshifted size-biased degrees

The graph $G_N$ may be constructed by joining the half-edges in a random order. In particular, $G_N$ may be constructed as the epidemic progresses; starting with the initial infected case we sequentially match the half-edges along which the disease is transmitted. Since half-edges are chosen uniformly at random in the matching procedure, the probability to choose a specific node is proportional to the number of free half-edges attached to the node in question. That is, if we pair a single half-edge, the probability of choosing a specific node with $k_s$ unpaired single half-edges is proportional to $k_s$. For this reason, the degree distribution of a node explored by joining a single half-edge in the early phase of the epidemic can be approximated by the *single size biased* [cf. the concept of excess degree in Meyers (2007)] degree distribution $p^{(s)}$

2 $$\begin{aligned} p^{(s)}(k_s, k_{\varDelta })=\frac{k_sp(k_s, k_{\varDelta })}{E(S)}. \end{aligned}$$

Similarly, the degree distribution of the nodes explored by joining three triangle half-edge pairs in the early phase of the epidemic can be approximated by the *triangle size biased* degree distribution $p^{(\varDelta )}$

3 $$\begin{aligned} p^{(\varDelta )}( k_s, k_{\varDelta })=\frac{k_{\varDelta } p(k_s, k_{\varDelta })}{E(\varDelta )}. \end{aligned}$$

In the epidemic process, we need to account for the fact that an infected individual has at least one non-susceptible neighbour (namely the direct source of its infection). For this reason, we introduce the *downshifted* size biased degree distributions $p^{(s)}_{\bullet }$ and $p^{(\varDelta )}_{\bullet }$, given by

4 $$\begin{aligned} p^{(s)}_{\bullet }(k_s, k_{\varDelta })= & {} p^{(s)}( k_s+1, k_{\varDelta })\nonumber \\ p^{(\varDelta )}_{\bullet }(k_s, k_{\varDelta })= & {} p^{(\varDelta )}(k_s, k_{\varDelta }+1). \end{aligned}$$

Throughout, we will make frequent reference to the following random vectors

5 $$\begin{aligned} \begin{array}{c} (S^{(s)}_{\bullet }, \varDelta ^{(s)}_{\bullet }) \sim p_{\bullet }^{(s)}\\ (S^{( \varDelta )}_{\bullet }, \varDelta ^{(\varDelta )}_{\bullet }) \sim p_{\bullet }^{(\varDelta )}\\ \end{array} \end{aligned}$$

and the expected values

6 $$\begin{aligned} E(S^{(s)}_{\bullet })&= \frac{E(S^2)}{E(S)}- 1 \quad E(\varDelta ^{(s)}_{\bullet }) =\frac{E(S\varDelta )}{E(S)}\nonumber \\ E(S^{( \varDelta )}_{\bullet })&=\frac{E(S\varDelta )}{E(\varDelta )}. \quad E(\varDelta ^{(\varDelta )}_{\bullet }) =\frac{E(\varDelta ^2)}{E(\varDelta )}- 1 \end{aligned}$$

### The epidemic model

We use an SIR model to investigate the dynamics of the spread of the disease. At any given time point, the population is divided into three groups, depending on health status. The groups are susceptible (**S**), infectious (**I**) and recovered (**R**) [see e.g. Britton (2010), Diekmann et al. (2013)]. Individuals of the population make contact with other individuals at (possibly random) points in time. If, at some time point, an infectious individual contacts a susceptible individual then the susceptible individual instantaneously becomes infectious. An infectious individual will cease to be contagious after a period of time, which we call the *infectious period* of the individual in question, and is then transferred to the recovered group. Recovered individuals are those that are immune to the disease. Individuals belonging to this group play no further role in the spread of the disease. Because of this last observation, we can treat individuals that die because of the disease as “recovered”. In summary, we allow only the transitions $S\rightarrow I$ and $I\rightarrow R$. Note that the population is assumed to be closed; we ignore births, deaths and migration.

More specifically, we consider an SIR epidemic in a generation framework on the clustered static graph $G_N$ and assume possible heterogeneity in infectivity, both between different individuals (individual heterogeneity) and between different kinds of edges (edge heterogeneity). Individual heterogeneity means that some infected individuals are more contagious than others. Such heterogeneity may, for instance, arise from variability in the infectious period. Edge heterogeneity reflects that individuals may exhibit different contact behaviors for different types of neighbours ; an individual may for instance prefer to spend more time with its triangle neighbours at the expense of spending less time with its single neighbours.

To construct a model that captures such heterogeneities, let $$T=(T_{s}, T_{\varDelta })$$ be a random variable with support in $$ [0,1]^2$$, and let $$\{T_i\}_{i=1}^N=\{(T_{s}^{(i)}, T_{\varDelta }^{(i)})\}_i$$ be a sequence of independent copies of *T*. We allow for any dependence structure between $$T_{s}$$ and $$T_{\varDelta }$$. Each node $$v_i$$ of $$G_N$$ is equipped with a two-dimensional *transmission weight*$$T_i$$. If $$v_i$$ gets infected, then each susceptible single neighbour (neighbour by virtue of a single edge) of $$v_i$$ gets infected by $$v_i$$ independently in the next generation with probability $$T_{s}^{(i)}$$, and each susceptible triangle neighbour (neighbour by virtue of a triangle edge) of $$v_i$$ gets infected by $$v_i$$ independently in the next generation with probability $$T_{\varDelta }^{(i)}$$ (conditioned on $$\{T_i\}_i$$). Node $$v_i$$ thereafter becomes recovered, playing no further role in the epidemic. An infected node transmits the disease independently of the transmissions from other infected nodes. An infected node does not, however, transmit the disease to its neighbours independently, unless the distribution of *T* is degenerate. Conditioned on the transmission weights $$\{T_i\}_i$$ and the structure of $$G_N$$, the number of single and triangle neighbours that an infected node $$v_i$$ makes (infectious) contact with while infectious has a binomial distribution with parameters $$(S_i, T_{s}^{(i)})$$ and $$(\varDelta _i, T_{\varDelta }^{(i)})$$, respectively.

The spread of this epidemic can be fully captured by a directed graph [see e.g. Pellis et al. (2012), Kenah and Miller (2011)]. To construct such directed graph from an undirected CMC graph $G_N$, we replace each undirected edge of $G_N$ by two parallel directed edges, pointing in the opposite direction. The weight of an edge $(v_i,v_j)$, which represents the (potential) transmission time from $v_i$ to $v_j$, is taken to be 1 if $v_i$ would make infectious contact with $v_j$ if infected, and $\infty$ otherwise. The individuals ultimately infected are then the individuals that can be reached from an initial case by following a path consisting of directed edges with finite edge weights.

### Reproduction numbers

A key quantity in the study of epidemics is the basic reproduction number, often denoted by $R_0$. It is usually defined as the expected number of infected cases caused by a “typical” infected individual in an otherwise susceptible population (Diekmann et al. 1990). For most stochastic epidemic models [including SIR epidemics in homogeneous mixing populations (Britton 2010), populations with households (Ball et al. 2016) and epidemics on networks (Britton et al. 2007)] it has the threshold property that a major outbreak is possible if and only if $R_0>1$.

For models where a suitable generation based branching process approximation is available, $R_0$ is usually defined as the Perron root (the dominant eigenvalue, which exists and is real-valued by assumptions A1 and A2, see for instance Varga (2009, Chapter 2) of the mean matrix of the approximating Galton Watson branching process. This is the definition used in this article. By standard branching process theory, the interpretation of $R_0$ as the expected number of cases caused by the typical individual in the early phase of the epidemic and its threshold properties are retained by this definition. The threshold property of $R_0$ is made precise in Theorem 1 below.

In Sect. 4, we investigate the spread of an epidemic in a population with vaccination. To this end, in addition to the basic reproduction number $R_0$, we consider the *perfect vaccine-associated reproduction number*$R_V$ (Goldstein et al. 2009). A vaccine is *perfect* if it provides full and permanent immunity. That is, an individual vaccinated with a perfect vaccine cannot contract the disease. The perfect vaccine-associated reproduction number $R_V$ is defined as (Ball et al. 2016)

7 $$\begin{aligned} R_V=\frac{1}{1-f_{\text{ v }}^{(c)}}, \end{aligned}$$

where the *critical vaccination coverage*$f_{\text {v}}^{(c)}$ is the fraction of the population that has to be vaccinated with a perfect vaccine in order to reduce $R_0$ to unity, if the vaccinated individuals are chosen uniformly at random. That is to say, $f_{\text {v}}^{(c)}=1-1/R_V$ is the fraction necessary to vaccinate in order to be guaranteed to prevent a major outbreak (Britton 2010). Note that if $R_0\le 1$ then $f_{\text{ v }}^{(c)}=0.$

For many models, including epidemics on graphs generated by the CM (Britton et al. 2007) and the standard stochastic SIR epidemic model [i.e. individuals mix homogeneously, see for instance Britton (2010)], $R_V=R_0$. That is, vaccinating a fraction $1-1/R_0$ of the population with a perfect vaccine is sufficient to surely prevent a major outbreak. On the other hand, for the households and households-workplaces model with uniform vaccination, $R_V\ge R_0$ (Ball et al. 2016) with strict inequality possible. In Sect. 4.1 we show that for the model analysed in this report, $R_V=R_0.$

#### Epidemics in continuous time: the rank-based approach

As mentioned above, heterogeneity in infectivity might arise from heterogeneity in the infectious period; an important special case of the above described model is epidemics in continuous time with random infectious periods where contacts between individuals take place according to point processes on $\mathbb {R}_{\ge 0}$. Ignoring the real time-dynamics of an epidemic does not impact results that concern the final outcome of the epidemic. This result was first presented by Ludwig (1975), see also Pellis et al. (2008) or Kiss et al. (2017, section 6.2.3) for a more recent discussion. This leads us to the often more tractable *rank-based* approach.

**Fig. 2:**
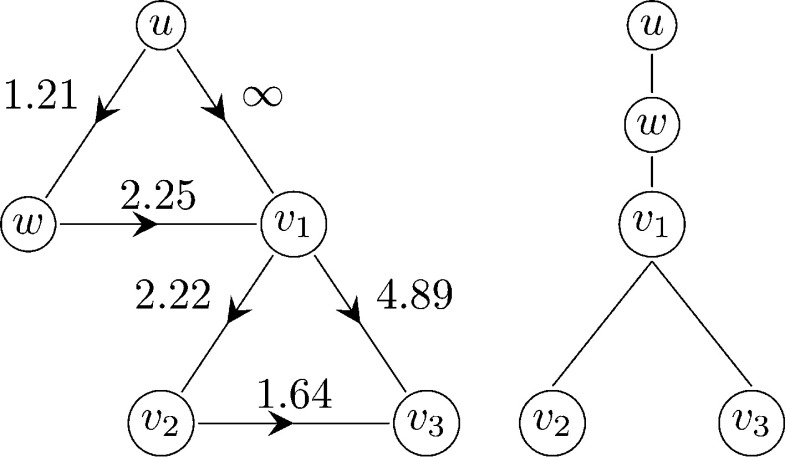
The difference between rank-based generations and true generations. Left: The length of the path $v_1\rightarrow v_3$ (i.e. the transmission time from $v_1$ to $v_3$) is 4.89 and exceeds the length $2.22+1.64$ of the path $v_1\rightarrow v_2\rightarrow v_3.$ Therefore, the true path of transmission is $u\rightarrow w\rightarrow v_1\rightarrow v_2\rightarrow v_3.$ In the rank-based approach, however, $v_3$ is attributed to $v_1$. Right: The resulting rank generation tree

In order to define the rank of a vertex, denote the initial case by $v_*$. The *rank* of a node *v* in $G_N$ is the distance from $v_ *$ to *v*, if every edge along which the disease would be transmitted is assigned the edge weight 1, and every other edge is assigned the edge weight $\infty$. That is, the rank of *v* is the smallest number of directed edges that have to be traversed in order to follow a path of (potential) transmission from $v_*$ to *v*. We may then analyse the spread of the disease by letting generation *n* of the epidemic process consist of the individuals of rank *n*. If, for instance, $v_1$ is the first node in a triangle consisting of the nodes $v_1,v_2,v_3$ to be infected, and $v_1$ infects $v_2$ and thereafter attempts to infect $v_3$, then $v_3$ is attributed to $v_1$ regardless of whether $v_1$ or $v_2$ infected $v_3$. This is illustrated in Fig. 2.

Consider a continuous time epidemic formulated as follows. Suppose that each infected individual remains infectious for a (random) period of time. The infectious periods are distributed as the random variable $\tau$, $\tau \sim F$, and independent (but identically distributed) for different nodes. Suppose further that a node makes contact with each neighbour independently at a Poisson rate while infected, and that susceptible individuals are fully susceptible, so that each infectious-susceptible contact results in transmission. If the contact rate is given by $\beta _s$ for single edge neighbours and $\beta _{\varDelta }$ for triangle edge neighbours then the transmission weights $T_{s}$ and $T_{\varDelta }$ are distributed as $1-e^{-\beta _s \tau }$ and $1-e^{-\beta _{\varDelta } \tau }$, respectively. We then have $E(T_{s})=1-\mathcal {L}(\beta _s)$, $E(T_{\varDelta })=1-\mathcal {L}(\beta _{\varDelta })$ and $E(T_{\varDelta }(1-T_{\varDelta }))= \mathcal {L}(\beta _{\varDelta })-\mathcal {L}(2\beta _{\varDelta })$ where $\mathcal {L}(z)=\int _{\mathbb {R}_+}e^{-z x}dF(x)$ is the Laplace transform of the infectious period.

### Branching process approximations

To analyse the spread of the disease in the early stages of the epidemic, we employ a multi-type branching process approximation. The graph $G_N$ may be constructed by joining the half-edges in any (possibly random) order, provided that the uniform matching is not violated. In particular, the graph $G_N$ may be constructed (or explored) as the epidemic progresses; starting with the initial infected case $u^{*}$ we sequentially match the half-edges along which the disease is transmitted. In the early phase of the epidemic, short cycles (except for the triangles formed by triangle edges) are unlikely to appear. For these reasons, the early spread of the disease is well approximated by a suitably chosen branching process.

Similarly, a branching process approximation can be used to approximate the expected final size of the epidemic (Ball et al. 2009, 2010, 2014). In the graph representation of an epidemic, an individual contracts the disease if and only if there is a path of directed edges with finite edge weights from the initial case to the node representing the individual in question.

**Fig. 3:**
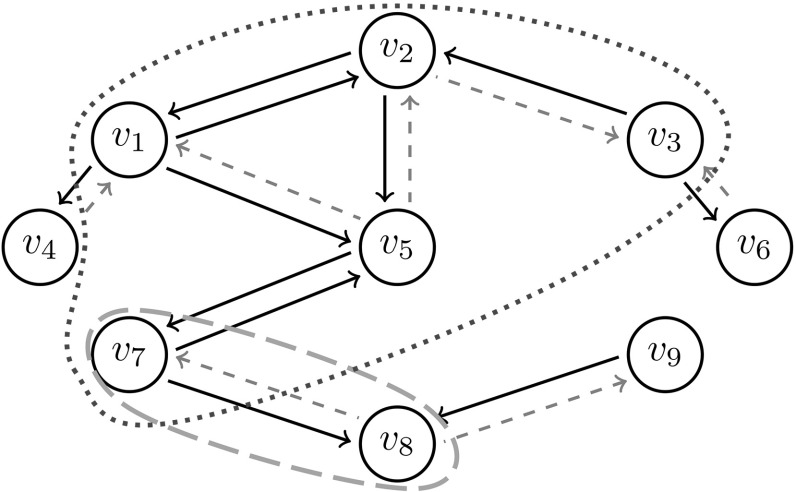
Graph representation of an epidemic in a small $(N=9)$ population. The gray dashed and black solid edges have infinite and finite edge weights (transmission times), respectively. The nodes in the susceptibility set of $v_5$, $\mathcal {S}(v_5) =\{v_1.v_2, v_3,v_5,v_7\}$, are enclosed by the blue dotted line. The nodes that $v_5$ would infect if infected, directly or through other nodes, are enclosed by the orange dashed line (colour figure online)

Define the susceptibility set $\mathcal {S}(v)=\mathcal {S}_N(v)$ of a node *v* as the collection of nodes of $G_N$ that can be reached from *v* by tracing a path of finite length backwards. That is, the individuals that contract the disease are precisely the individuals with susceptibility sets that contain an initial case. Hence, if the initial case is chosen uniformly at random then the probability that a node *v* contracts the disease is proportional to the size of its susceptibility set $\mathcal {S}(v)$ and this probability can be approximated by exploring $\mathcal {S}(v)$. Figure 3 shows a schematic illustration of a susceptibility set.

By reversing the direction of the edges of the graph representation of an epidemic, but keeping the weights, the expected final fraction of the population infected in a major outbreak and the probability of a major outbreak are interchanged (Miller 2008), provided that the initial case is chosen uniformly at random. The process so obtained is called the *backward epidemic process* of the node *v*. If the underlying epidemic model is such that the backward epidemic process can be well approximated by a branching process, then we can use this branching process to compute the asymptotic distribution of the proportion of the population that ultimately escapes infection. This is made precise in the following theorem, due to Ball et al. (2014, Theorem 3.5), who proved the theorem for the related model of random intersection graphs. The statement of Theorem 1 carries over to the forward and backward branching processes considered in this paper. We omit the proof, which is analogous to the proof presented in Ball et al. (2014), see also Ball et al. (2009).

#### Theorem 1

Let *q* and $q_b$ be the extinction probabilities of the forward and backward approximating branching processes respectively, and let $S_N$ be the proportion of the population that ultimately escapes an epidemic in a population of size *N*. Then

8 $$\begin{aligned} S_N\rightarrow S \end{aligned}$$

as $N\rightarrow \infty$ where the convergence in (8) is in distribution and $P(S = 1)=1-P(S = q_b)=q$.

In other words, in the limit of large population sizes, the epidemic “takes off” with probability $$ 1-q$$, and if this happens a fraction $$ 1-q_b$$ of the population is ultimately infected (with probability converging to 1 as $$N \rightarrow \infty $$). Note that since $$R_0$$ is defined as the Perron root of the mean matrix of the forward branching process, $$q<1$$ if and only if $$R_0>1$$.

## An epidemic in a fully susceptible population

We now have the tools to analyse the spread of an infectious disease on a graph generated by the CMC. In the present section, the population is assumed to be fully susceptible to the disease, apart from the initially infectious individual.

### Forward process

Before analysing the forward process, we need to set some terminology. For a given triangle *u*, *v*, *w*, where *u* is the first individual to be infected in the triangle *u*, *v*, *w*, we refer to *v* and *w* as *twins.* We approximate the spread of the disease during the early phase by a multi-type branching process consisting of the following three types (except for the initial case):

Type 1: A node infected along a triangle edge whose twin (in the same triangle) is infected at the same time step or earlier
Type 2: A node infected along a triangle edge that is not of type 1
Type 3: A node infected along a single edge

**Fig. 4:**
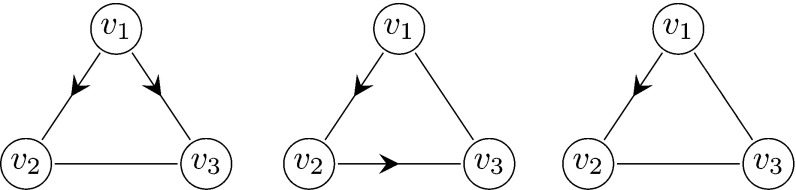
Three examples of possible paths of transmission in a triangle $v_1,v_2,v_3$, where $v_1$ is the first node to be infected. Left: $v_1$ infects both $v_2$ and $v_3$. Both $v_2$ and $v_3$ are represented by type 1 individuals in the approximating branching process. Center: $v_1$ infects $v_2$ and $v_2$ infects $v_3$. Then $v_3$ and $v_2$ are represented by type 1 and type 2 individuals, respectively. Right: $v_1$ infects $v_2$. Then $v_2$ is represented by a type 2 individual

Figure 4 shows three examples of possible paths of transmission within a triangle giving rise to type 1 and 2 individuals in the approximating branching process.

Denote by

$$\begin{aligned} M_f=(m_{i,j})_{i,j=1}^3 = \begin{pmatrix} m_{1,1} &{}\quad m_{1,2} &{}\quad m_{1,3}\\ m_{2,1} &{}\quad m_{2,2} &{}\quad m_{2,3}\\ m_{3,1} &{}\quad m_{3,2} &{}\quad m_{3,3}\\ \end{pmatrix} \end{aligned}$$

the mean matrix of the above described branching process. Suppose that $$v_1$$ is the first individual to be infected in the triangle $$v_1$$, $$v_2$$, $$v_3$$. The probability that $$v_1$$ transmits the disease both to $$v_2$$ and $$v_3$$ is $$E(T^2). $$ Similarly, the probability that $$v_1$$ transmits the disease to either $$v_2$$ or $$v_3$$, but not to both, is $$2E(T(1-T)). $$

Thus, by linearity of expectation and because the distribution of the susceptible neighbours of infected nodes in the early phase of the epidemic is given by the downshifted degree distributions in (4), we obtain

9 $$\begin{aligned} M_f=\begin{pmatrix} 2E(T_{\varDelta }^2)E(\varDelta _{\bullet }^{(\varDelta )}) &{} \quad 2E(T_{\varDelta }(1-T_{\varDelta }))E(\varDelta _{\bullet }^{(\varDelta )})&{}\quad E(T_{s})E(S_{\bullet }^{(\varDelta )})\\ 2E(T_{\varDelta }^2)E(\varDelta _{\bullet }^{(\varDelta )})+E(T_{\varDelta }) &{}\quad 2E(T_{\varDelta }(1-T_{\varDelta }))E(\varDelta _{\bullet }^{(\varDelta )}) &{}\quad E(T_{s})E(S_{\bullet }^{(\varDelta )}) \\ 2E(T_{\varDelta }^2)E(\varDelta _{\bullet }^{(S)}) &{}\quad 2E(T_{\varDelta }(1-T_{\varDelta }))E(\varDelta _{\bullet }^{(S)}) &{}\quad E(T_{s})E(S_{\bullet }^{(S)})\\ \end{pmatrix}. \end{aligned}$$

(Recall that the random variables $\varDelta _{\bullet }^{(\varDelta )}$, $\varDelta _{\bullet }^{(s)}$, $S_{\bullet }^{(\varDelta )}$ and $S_{\bullet }^{(s)}$ defined in (5) have the downshifted size biased distributions). Note that all entries of $M_f$ are finite and that *S* and $\varDelta$ both have finite second moments by assumption A1.

If $M_f$ is positively regular (see the last paragraph before Sect. 2.1.1) then $R_0$ is given by the Perron root of $M_f$. With little effort, one can use the expected values provided in (6) to show that necessary and sufficient conditions for $M_f$ to be positively regular are that assumptions A1-A2 hold and that $0<E(T_{s})<1$ and $0<E(T_{\varDelta })<1$. If some of these conditions are not satisfied, we may analyse the spread of the disease by reducing the number of types of the approximating forward branching process. It is worth pointing out that $R_0$ only depends on the marginal distributions of $T_{s}$ and $T_{\varDelta }$ (via their moments), not on the dependence structure between them.

#### Probability of a major outbreak

For two *s*-dimensional vectors $\bar{a}=(a_1,\ldots , a_s)^{\mathsf {T}}$ and $\bar{b}=(b_1,\ldots , b_s)^{\mathsf {T}}$, we define

$$\begin{aligned} \bar{a}^{ \bar{b}}:= a_1^{b_1}\cdot \ldots \cdot a_s^{b_s}. \end{aligned}$$

Let $f:[0,1]^3\rightarrow \mathbb {R}^3$ be the probability generating function of the offspring distribution of the three types in the approximating branching process. That is, for $\bar{z}=(z_1,z_2,z_3)^{\mathsf {T}}\in [0,1]^3$ the *i*th component of $f(\bar{z})$ is given by

10 $$\begin{aligned} f(\bar{z})_i=E\left( \bar{z}^{ \bar{\xi }_i}\right) \end{aligned}$$

where $\bar{\xi }_{i}=(\xi _{i,1}, \xi _{i,2},\xi _{i,3})$ is distributed as the offspring of a type *i* individual, $i=1,2,3.$

Similarly, let $f_*:[0,1]^3\rightarrow \mathbb {R}$ be the probability generating function of the offspring distribution of the initial case. If $\bar{\xi }=(\xi _{*,1},\xi _{*,2},\xi _{*,3})^{\mathsf {T}}$ is distributed as the offspring of the initial case, then $f_*$ is given by

$$\begin{aligned} f_*(\bar{z})=E\left( \bar{z}^{\bar{\xi }}\right) . \end{aligned}$$

For $i=1,2,3$, let $(S^{(i)}, \varDelta ^{(i)})$ be the joint degree of a type *i* case with offspring $(\xi _{i,1}, \xi _{i,2},\xi _{i,3})$ and transmission weight $T=(T_{s},T_{\varDelta })$. That is,

$$\begin{aligned} (S^{(1)}, \varDelta ^{(1)})\overset{d}{=}(S^{(2)}, \varDelta ^{(2)})\overset{d}{=}(S^{(\varDelta )}, \varDelta ^{(\varDelta )}) \end{aligned}$$

and

$$\begin{aligned} (S^{(3)}, \varDelta ^{(3)})\overset{d}{=}(S^{(s)}, \varDelta ^{(s)}). \end{aligned}$$

Here $\overset{d}{=}$ denotes equality in distribution. By conditional independence we have

$$\begin{aligned} E(z_1^{\xi _{i,1}}z_2^{\xi _{i,2}}z_3^{\xi _{i,3}})=E\left( E(z_3^{\xi _{i,3}}\vert T,S^{(i)},\varDelta ^{(i)})E(z_1^{\xi _{i,1}}z_2^{\xi _{i,2}}\vert T,S^{(i)},\varDelta ^{(i)})\right) . \end{aligned}$$

Conditioned on the transmission weight *T* and the single degree $S^{(1)}$, $\xi _{1,3}$ has a binomial distribution with parameters $S^{(1)}$ and $T_{s}$. Thus

$$\begin{aligned} E(z_{3}^{\xi _{1,3}}\vert T,S^{(1)},\varDelta ^{(1)})= & {} \sum _{k_0+k_1=S^{(1)}}{{S^{(1)}}\atopwithdelims (){k_1} } (T_{s}z_3)^{k_1}(1-T_{s})^{k_0}\\= & {} (T_{s}z_3+1-T_{s})^{S^{(1)}}. \end{aligned}$$

Similarly

$$\begin{aligned}&E(z_1^{\xi _{1,1}}z_2^{\xi _{1,2}}\vert T,S^{(1)},\varDelta ^{(1)})\\&\quad =\sum _{k_0+k_1+k_2=\varDelta ^{(1)}-1}{{\varDelta ^{(1)}-1} \atopwithdelims (){k_0,k_1,k_2} }(1-T_{\varDelta })^{2k_0} (2(1-T_{\varDelta }) T_{\varDelta }z_2)^{k_1}(T_{\varDelta }z_1)^{2k_2}\\&\quad =((1-T_{\varDelta })^2+2T_{\varDelta }(1-T_{\varDelta })z_2+T_{\varDelta }2z_1^2)^{\varDelta ^{(1)}-1}. \end{aligned}$$

Thus

11 $$\begin{aligned}&E(z_1^{\xi _{1,1}}z_2^{\xi _{1,2}}z_3^{\xi _{1,3}})\nonumber \\&\quad =E((T_{s}z_3+1-T_{s})^{S_{\bullet }^{(\varDelta )}}((1-T_{\varDelta })^2 +2T_{\varDelta }(1-T_{\varDelta })z_2+T_{\varDelta }^2z_1^2)^{\varDelta _{\bullet }^{(\varDelta )}}) \end{aligned}$$

where $(\varDelta _{\bullet }^{(\varDelta )}, S_{\bullet }^{(\varDelta )})$ is independent of *T*.

Since the conditional offspring distribution of a type 2 individual is identical to the offspring distribution of a type 1 individual except that a type 2 individual may give birth to one additional type 1 individual with probability $T_{\varDelta }$, we have

12 $$\begin{aligned}&E(z_1^{\xi _{2,1}}z_2^{\xi _{2,2}}z_3^{\xi _{2,3}})\nonumber \\&\quad =E((T_{s}z_3+1-T_{s})^{S_{\bullet }^{(\varDelta )}}((1-T_{\varDelta })^2\nonumber \\&\qquad +\,2T_{\varDelta }(1-T_{\varDelta })z_2+T_{\varDelta }^2z_1^2)^{\varDelta _{\bullet }^{(\varDelta )}} (T_{\varDelta }z_1+1-T_{\varDelta })). \end{aligned}$$

Similarly,

13 $$\begin{aligned}&E(z_1^{\xi _{3,1}}z_2^{\xi _{3,2}}z_3^{\xi _{3,3}})\nonumber \\&\quad = E((T_{s}z_3+1-T_{s})^{S_{\bullet }^{(s)}}((1-T_{\varDelta })^2 +2T_{\varDelta }(1-T_{\varDelta })z_2+T_{\varDelta }^2z_1^2)^{\varDelta _{\bullet }^{(s)}}). \end{aligned}$$

Substituting (11)–(13) into (10) gives an expression for *f*.

By standard branching process theory, if $$R_0>1$$ the extinction probability of a process descending from a type *i* individual, $$i=1,2,3,$$ is given by $$q_i$$, where $$\bar{q}=(q_1,q_2,q_3)^{\mathsf {T}}$$ is the unique solution of $$\bar{q}=f(\bar{q})$$ in $$[0,1)^3$$. We also have

14 $$\begin{aligned} \bar{q}=\lim _{n\rightarrow \infty } f^{\circ n}(\bar{0}), \end{aligned}$$

where $f^{\circ n}$ is the composition of *f* with itself *n* times.

Since the approximating branching process dies out if and only if each of the processes started by the children of the initial case die out, the probability of extinction is given by $f^{*}(\bar{q}).$ After some calculations, analogous to the calculations that led to (11)–(13), we find that the probability of extinction is given by

$$\begin{aligned} f^{*}(\bar{q})=E\left( (T_{s}q_3+1-T_{s})^{S}((1-T_{\varDelta })^2+2T_{\varDelta }(1-T_{\varDelta })q_2 +T_{\varDelta }^2q_1^2)^{\varDelta }\right) \end{aligned}$$

where $$(S, \varDelta )$$ is independent of *T*. We conclude that, by Theorem 1, the probability of a major outbreak is given by $$1-f^{*}(\bar{q}),$$ where $$\bar{q}$$ is the limit in (14) and also the fixed point of *f* in $$[0,1)^3$$.

### Backward process

Let *w* be a given node of $G_N$, chosen uniformly at random. We use a backward branching process to approximate the probability that *w* contracts the disease, which by an exchangeability argument equals the expected final size of a major outbreak. The offspring of an individual *v* in the backward process are the individuals that would potentially have infected *v*, if they were infected themselves.

The members of the susceptibility set are divided into the following two groups (which give rise to a two-type approximating backward branching process).

Type 1: The vertex is included in the susceptibility set by virtue of potential transmission along a single edge
Type 2: The vertex is included in the susceptibility set by virtue of potential transmission along a triangle edge

We assign kinship as follows. The children of type 1 of an individual $v_1$ are the individuals included in the susceptibility set due to potential transmission along a single edge. The children of type 2 of $v_1$ are the individuals included in the susceptibility set due to potential transmission of the disease to $v_1$, within a triangle of which $v_1$ is a member. We note that, given a triangle $v_1,v_2, v_3$ where $v_1$ is the primary case, both $v_2$ and $v_3$ will be members of the susceptibility set of $v_1$ by virtue of transmissions within the triangle if at least one of the following events happens:

($$E_{1}$$)
$v_2$ and $v_3$ both “infect” $v_1$
($$E_{2}$$)
$v_2$ infects $v_1$ and $v_3$ “infects” $v_2$
($$E_{3}$$)
$v_3$ infects $v_1$ and $v_2$ “infects” $v_3$

Here “infects” is conditional on the “infector” being infected during the epidemic.

**Fig. 5:**
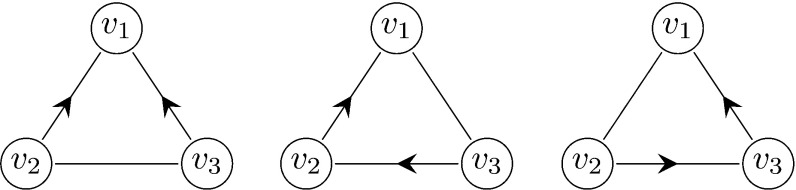
The individuals $v_2$ and $v_3$ are both in the susceptibility set $\mathcal {S}(v_1)$ of $v_1$ by virtue of transmission within the triangle $v_1,v_2,v_3$ if and only if at least one of the events $E_{1}$ (left), $E_{2}$ (center) or $E_{3}$ (right) happens

The events $E_{1}$-$E_{3}$ are illustrated in Fig. 5.

Standard calculations give that the probability of the union of the events $E_{1}$-$E_{3}$ is given by $p_2=3E(T_{\varDelta })^2-2E(T_{\varDelta })E(T_{\varDelta }^2)$. Similarly, the probability that neither $v_1$ nor $v_2$ will be members of the susceptibility set of *v* by transmissions within the triangle is given by $p_0=(1-E(T_{\varDelta }))^2$. For later use, denote $1-p_0-p_2$ by $p_1$.

#### Expected final size of a major outbreak

Let *b* be the probability generating function of the offspring distribution of the two types of the approximating backward branching process. Furthermore, let $$b_*$$ be the probability generating function of the offspring distribution of the ancestor *w*. Analogously to the forward branching process, the probability that a branching population whose ancestor is of type $$i,\ i=1,2,$$ will go extinct is given by $$q^b_i$$, where $$\bar{q}_b=(q_1^b,q_2^b)^{\mathsf {T}}$$ is the unique solution of $$\bar{q}_b=b(\bar{q}_b)$$ in $$[0,1)^2$$ (recall $$R_0>1$$). The probability of extinction is given by $$b_*(\bar{q}_b)$$.

Proceeding in the same manner as in Sect. 3.1.1 yields

$$\begin{aligned} b(z_1,z_2)_1=E\left( (E(T_{s})z_1+1-E(T_{s}))^{S_{\bullet }^{(s)}} (p_0+p_1z_2+p_2z_2^2)^{\varDelta _{\bullet }^{(s)}}\right) \end{aligned}$$

where $p_0,\ p_1$ and $p_2$ are as in Sect. 3.2. Similarly

$$\begin{aligned} b(z_1,z_2)_2= E\left( (E(T_{s})z_1+1-E(T_{s}))^{S_{\bullet }^{(\varDelta )}} (p_0+p_1z_2+p_2z_2^2)^{\varDelta _{\bullet }^{(\varDelta )}}\right) , \end{aligned}$$

and the probability of ultimate extinction of the backward process is given by

$$\begin{aligned} b_*(\bar{q}_ b)=E\left( (E(T_{s})q_1^b+1-E(T_{s}))^{S} (p_0+p_1q^b_2+p_2(q_2^b)^2)^{\varDelta }\right) . \end{aligned}$$

We conclude that the expected final size of a major outbreak is given by $1-b_*(\bar{q}_ b).$

## Vaccination

### Random vaccination with a perfect vaccine

Assume that a fraction $f_{\text{ v }}<1$ of the population is vaccinated, and that the vaccinated individuals are chosen uniformly at random (without replacement) from the population. The vaccine is perfect, in the sense that a vaccinated individual gains full and lasting immunity to the disease. If the population size *N* is large, we may use a slightly different model, where each individual is vaccinated with probability $f_{\text{ v }}$, independently of the vaccination status of other individuals. By the law of large numbers, for our purposes the models are equivalent in the limit as the population size $N\rightarrow \infty .$

As before, we may approximate the early phase of the epidemic by a multi-type branching process. The individuals of the approximating branching process are now of the following three types.

Type 1: Infected along a triangle edge and has a twin that is known not to be susceptible
Type 2: Infected along a triangle edge and has a twin that might be susceptible
Type 3: Infected along a single edge

To clarify the types, assume that in the early phase of the epidemic $v_1$ is the primary case in the triangle $v_1, v_2,v_3$. If $v_1$ attempts to transmit the disease both to $v_2$ and $v_3$ and succeeds (that is, none of $v_2$ and $v_3$ are vaccinated) then both $v_2$ and $v_3$ are represented by type 1 individuals in the approximating branching process. This happens with probability

15 $$\begin{aligned} E(T_{\varDelta }^2)(1-f_{\text{ v }})^2. \end{aligned}$$

If $v_1$ attempts to transmit the disease both to $v_2$ and $v_3$, but only succeeds to transmit the disease to $v_3$ (that is, $v_2$ is vaccinated and $v_3$ is not vaccinated), then in the approximating branching process the individual representing $v_1$ gives birth to one type 1 individual (representing $v_3$) within the triangle $v_1,v_2, v_3$. This happens with probability

16 $$\begin{aligned} E(T_{\varDelta }^2)f_{\text{ v }}(1-f_{\text{ v }}). \end{aligned}$$

If $v_1$ attempts to transmit the disease only to $v_2$ and succeeds (that is, $v_2$ is not vaccinated) then in the approximating branching process, the individual representing $v_1$ gives birth to one type 2 individual (representing $v_2$) within the triangle $v_1,v_2,v_3$. This happens with probability

17 $$\begin{aligned} E(T_{\varDelta }(1-T_{\varDelta }))(1-f_{\text{ v }}). \end{aligned}$$

**Fig. 6:**
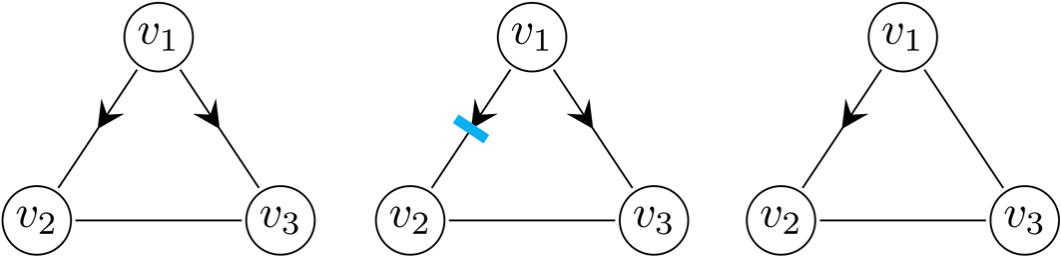
Three examples of transmission dynamics within a triangle $v_1,v_2,v_3$. An attempted transmission of the disease is represented by an arrow, an attempted transmission to a vaccinated individual is represented by an arrow and a blue bar. Left: $v_1$ attempts to transmit the disease both to $v_2$ and $v_3$, and succeeds. Both $v_2$ and $v_3$ are represented by type 1 individuals in the approximating branching process. Center: $v_1$ attempts to transmit the disease both to $v_2$ and $v_3$, the transmission to $v_2$ is blocked since $v_2$ is vaccinated. Then $v_3$ is represented by a type 1 individual. Right: $v_1$ succeeds to transmit the disease to $v_2$, but does not attempt to infect $v_3$. Then $v_2$ is represented by a type 2 individual (colour figure online)

The above described events are illustrated in Fig. 6.

Denote the mean matrix of the approximating branching process by $M_ f^{(\text{ v })}=(m_{i,j}^{(\text{ v })})_{i,j=1}^3$. Using the expressions in (15) and (16) gives the expected number of type 1 individuals produced by a type 1 individual

$$\begin{aligned} m_{1,1}^{(\text{ v })}= & {} \left( 2(1-f_{\text{ v }})^2E(T_{\varDelta }^2) +2(1-f_{\text{ v }})f_{\text{ v }}E(T_{\varDelta }^2)\right) E \left( \varDelta _{\bullet }^{(\varDelta )}\right) \\= & {} (1-f_{\text{ v }})2E(T_{\varDelta }^2)E(\varDelta _{\bullet }^{(\varDelta )})\\= & {} (1-f_{\text{ v }})m_{1,1} \end{aligned}$$

where $m_{1,1}$ is an element of the mean matrix $M_f$ of the forward branching process presented in (9).

Proceeding in the same fashion, we obtain the elements of the mean matrix $M_ f^{(\text{ v })}=(m_{i,j}^{({\text{ v }})})_{i,j=1}^3$ of the branching process with random vaccination. It turns out that

$$\begin{aligned} M_ f^{(\text{ v })}=(1-f_{\text{ v }})M_f. \end{aligned}$$

It is readily verified that the Perron root of $M_f^{(\text{ v })}$ is

18 $$\begin{aligned} r_f^{(\text{ v })}=(1-f_{\text{ v }})r_f, \end{aligned}$$

where $r_f$ is the Perron root of $M_f$. Setting $r_f^{(\text{ v })}$ to 1 in (18) and solving for $f_{\text{ v }}$ yields the critical vaccination coverage $f_{\text{ v }}^{(c)}=1-1/r_f$.

We conclude that, for this particular graph model, equality holds between the basic reproduction number $R_0$ and the perfect vaccine-associated reproduction number $R_V$ as defined in (7). Note that $R_0$ is based on a rank-based perspective of infection and not on “who-infected-whom.

#### Probability of a major outbreak

Let *h* be the probability generating function of the offspring distribution of the three types in our model including vaccination. As in Sect. 3.1.1, we use the probability generating function to approximate the probability of extinction of the epidemic. To this end, let $$(\zeta _{i,1}, \zeta _{i,2},\zeta _{i,3})$$ be distributed as the offspring of a type *i* individual with transmission weight *T*, $$i=1,2,3, $$ and let $$(S^{(i)}, \varDelta ^{(i)})$$ be distributed as the joint degree of this individual. That is,

$$\begin{aligned} (S^{(1)}, \varDelta ^{(1)})\overset{d}{=}(S^{(2)}, \varDelta ^{(2)})\overset{d}{=}(S_{\circ }^{(\varDelta )}, \varDelta _{\circ }^{(\varDelta )}) \end{aligned}$$

and

$$\begin{aligned} (S^{(3)}, \varDelta ^{(3)})\overset{d}{=}(S_{\circ }^{(s)}, \varDelta _{\circ }^{(s)}). \end{aligned}$$

Note that $(S^{(i)}, \varDelta ^{(i)})$ and *T* are independent.

By conditional independence

$$\begin{aligned} E\left( z_1^{\zeta _{1,1}}z_2^{\zeta _{1,2}}z_3^{\zeta _{1,3}}\right) =E\left( E\left( z_3^{\zeta _{1,3}}\vert S^{(1)}, \varDelta ^{(1)}, T \right) E \left( z_1^{\zeta _{1,1}}z_2^{\zeta _{1,2}}\vert S^{(1)}, \varDelta ^{(1)}, T\right) \right) \end{aligned}$$

for $$\bar{z}=(z_1,z_2,z_3)^{\mathsf {T}}\in [0,1]^3$$.

Conditioned on the transmission weight *T* and the joint degree $(S^{(1)}, \varDelta ^{(1)})$, the number of attempted transmissions from a type 1 individual along single edges has a binomial distribution with parameters $S^{(1)}$ and $T_{s}$, and each attempted transmission succeeds with probability $(1-f_{\text{ v }})$. Thus,

19 $$\begin{aligned} E\left( z_3^{\zeta _{1,3}}\vert S^{(1)}, \varDelta ^{(1)}, T \right)= & {} \sum _{k_0+k_1=S^{(1)}} {S^{(1)} \atopwithdelims ()k_1}z_3^{k_1} \big (T_{s}(1-f_{\text{ v }})\big )^{k_1}\big ((1-T_{s})+T_{s}f_{\text{ v }}\big )^{k_0}\nonumber \\= & {} \big (T_{s}(1-f_{\text{ v }})z_3+1-T_{s}+T_{s}f_{\text{ v }}\big )^{S^{(1)}}. \end{aligned}$$

Similarly, for a type 1 individual *w* with triangle degree $\varDelta ^{(1)}$, by conditioning on the number of attempted transmissions (in $k_i$ of the $\varDelta ^{(1)}-1$ triangles that are not yet affected by the disease, *w* attempts to transmit the disease to *i* individuals, $i=0,1,2$) and the vaccination status of the individuals contacted by *w* we obtain

20 $$\begin{aligned}&E(z_1^{\zeta _{1,1}}z_2^{\zeta _{1,2}}\vert S^{(1)}, \varDelta ^{(1)}, T)\nonumber \\&\quad =\sum _{k_0+k_1+k_2=\varDelta ^{(1)}-1} {\varDelta ^{(1)}-1 \atopwithdelims ()k_0, k_1, k_2} (1-T_{\varDelta })^{2k_0}\big (2T_{\varDelta }(1-T_{\varDelta })\big )^{k_1}T_{\varDelta }^{2k_2}\nonumber \\&\qquad \left( \sum _{\tilde{k}_0+\tilde{k}_1+\tilde{k}_2=k_2} {k_2 \atopwithdelims ()\tilde{k}_0, \tilde{k}_1,\tilde{k}_2} \big ((1-f_{\text{ v }})z_1\big )^{2\tilde{k}_2} \big (2f_{\text{ v }}(1-f_{\text{ v }})z_1\big )^{\tilde{k}_1} f_{\text{ v }}^{2\tilde{k}_0}\right) \nonumber \\&\qquad \left( \sum _{ k_0'+ k_1'=k_1} {k_1 \atopwithdelims ()k_0', k_1'} (1-f_{\text{ v }})^{k_1'}z_2^{k_1'} f_{\text{ v }}^{k_0'}\right) \nonumber \\&\quad =\sum _{k_0+k_1+k_2=\varDelta ^{(1)}-1} {\varDelta ^{(1)}-1 \atopwithdelims ()k_0, k_1, k_2} (1-T_{\varDelta })^{2k_0}\big (2T_{\varDelta }(1-T_{\varDelta })\big )^{k_1}T_{\varDelta }^{2k_2}\nonumber \\&\qquad \big (\big ((1-f_{\text{ v }})z_1\big )^2+2f_{\text{ v }}(1-f_{\text{ v }})z_1 +f_{\text{ v }}^2\big )^{k_2} \nonumber \\&\qquad \big ((1-f_{\text{ v }})z_2+f_{\text{ v }}\big )^{k_1}\nonumber \\&\quad =\Big [(1-T_{\varDelta })^2 +2T_{\varDelta }(1-T_{\varDelta }) \big [(1-f_{\text{ v }})z_2+f_{\text{ v }}\big ] \nonumber \\&\qquad +\,T_{\varDelta }^2\big [\big ((1-f_{\text{ v }})z_1)^2+2f_{\text{ v }}(1-f_{\text{ v }})z_1 +f_{\text{ v }}^2\big ]\Big ]^{\varDelta ^{(1)}-1}. \end{aligned}$$

Combining (19) and (20) yields

21 $$\begin{aligned} E\left( z_1^{\zeta _{1,1}}z_2^{\zeta _{1,2}}z_3^{\zeta _{1,3}}\right)= & {} E\bigg [\Big (T_{s}(1-f_{\text{ v }})z_3+1-T_{s}+T_{s}f_{\text{ v }}\Big )^{S_{\bullet }^{(\varDelta )}}\nonumber \\&\Big ((1-T_{\varDelta })^2 +2T_{\varDelta }(1-T_{\varDelta }) \big ((1-f_{\text{ v }})z_2+f_{\text{ v }}\big )\nonumber \\&\quad +\,T_{\varDelta }^2\big (\big ((1-f_{\text{ v }})z_1\big )^2+2f_{\text{ v }} (1-f_{\text{ v }})z_1+f_{\text{ v }}^2\big )\Big )^{\varDelta _{\bullet }^{(\varDelta )}}\bigg ].\qquad \end{aligned}$$

By noting that the offspring distribution of a type 2 individual is identical to the offspring distribution of a type 1 individual, except that a type 2 may give birth to one additional type 1 individual with probability $T_{\varDelta }(1-f_{\text{ v }})$ we obtain

22 $$\begin{aligned} E\left( z_1^{\zeta _{2,1}}z_2^{\zeta _{2,2}}z_3^{\zeta _{2,3}}\right)= & {} E\bigg [\Big (T_{s}(1-f_{\text{ v }})z_3+1-T_{s}+T_{s}f_{\text{ v }}\Big )^{S_{\bullet }^{(\varDelta )}}\nonumber \\&\Big ((1-T_{\varDelta })^2 +2T_{\varDelta }(1-T_{\varDelta }) \big ((1-f_{\text{ v }})z_2+f_{\text{ v }}\big )\nonumber \\&\quad +\,T_{\varDelta }^2\big (\big ((1-f_{\text{ v }})z_1\big )^2 +2f_{\text{ v }}(1-f_{\text{ v }})z_1 +f_{\text{ v }}^2\big )\Big )^{\varDelta _{\bullet }^{(\varDelta )}}\nonumber \\&\Big (z_1T_{\varDelta }(1-f_{\text{ v }})+1-T_{\varDelta }(1-f_{\text{ v }})\Big )\bigg ]. \end{aligned}$$

Similarly,

23 $$\begin{aligned} E\left( z_1^{\zeta _{3,1}}z_2^{\zeta _{3,2}}z_3^{\zeta _{3,3}}\right)= & {} E\bigg [\Big (T_{s}(1-f_{\text{ v }})z_3+1-T_{s}+T_{s}f_{\text{ v }}\Big )^{S_{\bullet }^{(s)}}\nonumber \\&\Big ((1-T_{\varDelta })^2 +2T_{\varDelta }(1-T_{\varDelta })\big ((1-f_{\text{ v }})z_2 +f_{\text{ v }}\big )\nonumber \\&\quad +\,T_{\varDelta }^2\big (\big (\big ((1-f_{\text{ v }})z_1)^2 +2f_{\text{ v }}(1-f_{\text{ v }})z_1 +f_{\text{ v }}^2\big )\Big )^{\varDelta _{\bullet }^{(s)}}\bigg ].\qquad \end{aligned}$$

Combining these results yields the probability generating function *h* of the offspring distribution of a type 1, 2, 3 individual respectively. That is, $h(\bar{z})_1$ is given by (21), $h(\bar{z})_2$ is given by (22) and $h(\bar{z})_3$ is given by (23).

The probability generating function $h^{*}$ of the initial case is given by

24 $$\begin{aligned} h^{*}(\bar{z})= & {} E(z_1^{\zeta _{*,1}}z_2^{\zeta _{*,2}}z_3^{\zeta _{*,3}})\nonumber \\= & {} E\bigg [\Big (T_{s}(1-f_{\text{ v }})z_3+1-T_{s}+T_{s}f_{\text{ v }}\Big )^{S}\nonumber \\&\Big ((1-T_{\varDelta })^2 +2T_{\varDelta }(1-T_{\varDelta })\big ((1-f_{\text{ v }})z_2+f_{\text{ v }}\big )\nonumber \\&\quad +\,T_{\varDelta }^2\big (\big ((1-f_{\text{ v }})z_1\big )^2+2f_{\text{ v }}(1-f_{\text{ v }})z_1 +f_{\text{ v }}^2\big )\Big )^{\varDelta }\bigg ]. \end{aligned}$$

for $\bar{z}=(z_1,z_2,z_3)^{\mathsf {T}}\in [0,1]^3$, where $(S, \varDelta )$ is distributed as the joint degree of the initial case and independent of *T*. The probability of extinction of the approximating branching process is given by $h^{*}(\bar{q}^{\ (\text{ v })}),$ where $\bar{q}^{\ (\text{ v })}$ is given by the point in $[0,1]^3$ closest to the origin that satisfies $\bar{q}^{\ (\text{ v })}=h(\bar{q}^{\ (\text{ v })}).$ Thus, by Theorem 1 the probability of a major outbreak is $1-h_*(\bar{q}^{\ (\text{ v })}).$

#### The backward process

We now turn our attention to the backward process and final size of an epidemic in a population where a fraction $f_{\text{ v }}$ is vaccinated with a perfect vaccine. To this end, we introduce the following three types, where individuals are classified by their vaccination status and the type of the edge along which they would transmit the disease if infected.

Type 1: Transmits along triangle edge, no information on vaccination status is available
Type 2: Transmits along triangle edge and is known not to be vaccinated since it is successfully infected by its twin
Type 3: Transmits along single edge, no information on vaccination status is available

**Fig. 7:**
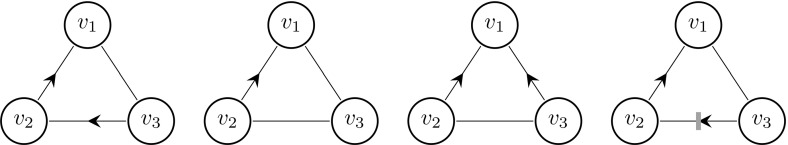
At least one of $v_2$ and $v_3$ will belong to the susceptibility set of $v_1$ by virtue of potential transmissions within the triangle if some of the following types of scenarios (left to right in the picture) occur: $E_{1}$, $E_{2}$, $E_{3}$, $E_{4}$. An attempted transmission of the disease is represented by an arrow, an attempted transmission to a vaccinated individual is represented by an arrow and a blue bar (colour figure online)

To clarify the types a bit more, let $v_1, v_2, v_3$ be a given triangle. At least one of $v_2$ and $v_3$ belongs to the susceptibility set of $v_1$ by virtue of potential transmissions within the triangle if some the following events, illustrated in Fig. 7, happens. Note that all cases infected by virtue of transmission within the triangle $v_1,v_2, v_3$ are attributed to $v_1$.

($$E_{1}$$)
$v_2$ attempts to infect $v_1$ and $v_3$ attempts to infect $v_2$, both succeed, and $v_3$ does not attempt to infect $v_1$. Or the same thing might happen, with $v_2$ and $v_3$ interchanged. This results in one type 1 and one type 2 individual in the approximating branching process. If $v_1$ is represented by a type 1 or 3 individual this happens with probability
  $$\begin{aligned} 2\big (1-f_{\text{ v }}\big )^2E(T_{\varDelta })E\big (T_{\varDelta }(1-T_{\varDelta })\big ), \end{aligned}$$
  if $v_1$ is represented by an individual of type 2 this happens with probability
  $$\begin{aligned} 2(1-f_{\text{ v }})E(T_{\varDelta })E\big (T_{\varDelta }(1-T_{\varDelta })\big ). \end{aligned}$$
($$E_{2}$$)
Only one of $v_2$ and $v_3$ attempts to infect $v_1$, and succeeds. The other node does not attempt to infect any node within the triangle. This results in one type 1 offspring. If $v_1$ is represented by an individual of type 1 or 3 this happens with probability
  $$\begin{aligned} 2(1-f_{\text{ v }})E(T_{\varDelta })E\big (T_{\varDelta }(1-T_{\varDelta })\big ), \end{aligned}$$
  if $v_1$ is represented by an individual of type 2 this happens with probability
  $$\begin{aligned} 2E(T_{\varDelta })E\big (T_{\varDelta }(1-T_{\varDelta })\big ). \end{aligned}$$
($$E_{3}$$)
$v_2$ and $v_3$ both attempt to infect $v_1$ and succeeds. This results in two type 1 individuals born in the approximating branching process. If $v_1$ is represented by an individual of type 1 or 3 this happens with probability
  $$\begin{aligned} (1-f_{\text{ v }})E(T_{\varDelta }^2), \end{aligned}$$
  if $v_1$ is represented by an individual of type 2 this happens with probability
  $$\begin{aligned} E(T_{\varDelta }^2). \end{aligned}$$
($$E_{4}$$)
$v_2$ attempts to infect $v_1$ and succeeds. The other node, $v_3$, attempts to infect $v_2$, but fails due to $v_2$ being vaccinated. The individual $v_3$ does not attempt to infect $v_1$. In this scenario, $v_2$ belongs to the susceptibility set of $v_1$. However, we do not include $v_2$ is the approximating branching process. This does not have any impact on the result of our analysis, since we are only interested in the probability of extinction of the backward process and $v_2$ does not produce any offspring in this process.

#### Expected final size

Let $b^{(\text{ v })}$ and $b^{(\text{ v })}_*$ be the probability generating function of the offspring distribution of the three types of the approximating backward branching process and of the ancestor, respectively. Furthermore, let $\bar{\zeta }_i=({\zeta ^b_{i,1}}, {\zeta ^b_{i,2}}, {\zeta ^b_{i,3}})$ be distributed as the offspring of a type $i,i=1,2, 3$, individual and denote by $E_s$ the conditional expectation given that the parent of $\zeta ^b_{i,1},\zeta ^b_{i,2}, \zeta ^b_{i,3}$ is susceptible. Let further $\bar{\zeta }_*=({\zeta ^b_{*,1}},{\zeta ^b_{*,2}}, {\zeta ^b_{*,3}})$ be distributed as the offspring of the ancestor. Denote the extinction probability of a process descending from a type *i* individual by $q_i^b$, $i=1,2,3$ and let $\bar{q}^b=(q_1^b,q_2^b, q_3^b)^{\mathsf {T}}$.

To find an expression for $b^{(\text{ v })}$, we first note that for $\bar{z}=(z_1,z_2,z_3)^{\mathsf {T}}$

25 $$\begin{aligned} E\left( \bar{z}^{ \bar{\zeta }_3}\right) =f_{\text{ v }}+(1-f_{\text{ v }})E_s\left( E_s\left( z_3^{\zeta _{3,3}^b}\vert S^{(3)}, \varDelta ^{(3)} \right) E_s\left( z_1^{\zeta _{3,1}^b} z_2^{\zeta _{3,2}^b}\vert S^{(3)}, \varDelta ^{(3)}\right) \right) \nonumber \\ \end{aligned}$$

where, as before, $(S^{(i)}, \varDelta ^{(i)})$ is distributed as the joint degree of a type *i* individual, $i=1,2,3.$

Now

26 $$\begin{aligned} E_s\left( z_3^{\zeta _{3,3}}\vert S^{(3)}, \varDelta ^{(3)}\right)= & {} \sum _{k_0+k_1=S^{(3)}-1} {S^{(3)}-1 \atopwithdelims ()k_0, k_1} z_3^{k_1}E(T_{s})^{k_1}E(1-T_{s})^{k_0}\nonumber \\= & {} \big (E(T_{s})z_3+1-E(T_{s})\big )^{S^{(3)}-1}. \end{aligned}$$

By conditioning on the number of triangles $k_2$ in which an event of type $E_{3}$ occurs, the number of triangles $k_1^a$ in which an event of type $E_{1}$ occurs, the number of triangles $k_1^b$ in which an event of type $E_{4}$ occurs and the number of triangles $k_1^c$ in which an event of type $E_{2}$ occurs we obtain

27 $$\begin{aligned}&E_s(z_1^{\zeta _{3,1}}z_2^{\zeta _{3,2}}\vert S^{(3)}, \varDelta ^{(3)})\nonumber \\&\quad =\sum _{k_0+k_1^a+k_1^b+k_1^c+k_2=\varDelta ^{(3)}}{\varDelta ^{(3)} \atopwithdelims ()k_0,k_1^a,k_1^b,k_1^c,k_2}E(1-T_{\varDelta })^{2k_0}\nonumber \\&\qquad \Big (2E(T_{\varDelta })E\big (T_{\varDelta }(1-T_{\varDelta })\big )(1-f_{\text{ v }})\Big )^{k_1^a}\nonumber \\&\qquad \Big (2E(T_{\varDelta })E\big (T_{\varDelta }(1-T_{\varDelta })\big )f_{\text{ v }}\Big )^{k_1^b} \Big (2E(T_{\varDelta })E\big ((1-T_{\varDelta })^2\big )\Big )^{k_1^c} \nonumber \\&\qquad E(T_{\varDelta })^{2k_2} z_2^{k_1^a}z_1^{k_1^a+k_1^c+2k_2}\nonumber \\&\quad =\Big (\big (E(1-T_{\varDelta })\big )^2+2E\big (T_{\varDelta }\big )E \big (T_{\varDelta }(1-T_{\varDelta })\big )(1-f_{\text{ v }})z_2z_1\nonumber \\&\qquad +\,2E(T_{\varDelta })E \big (T_{\varDelta }(1-T_{\varDelta })\big )f_{\text{ v }}\nonumber \\&\qquad +\,2E(T_{\varDelta })E\big ((1-T_{\varDelta })^2\big )z_1 +E(T_{\varDelta })^2z_1^2\Big )^{\varDelta ^{(3)}}. \end{aligned}$$

Inserting the right hand sides of (26) and (27) in (25) gives

28 $$\begin{aligned}&E(z_1^{\zeta _{3,1}}z_2^{\zeta _{3,2}}z_3^{\zeta _{3,3}})\nonumber \\&\quad =f_{\text{ v }}+(1-f_{\text{ v }})E \bigg [\Big (E(T_{s})z_3+1-E(T_{s})\Big )^{S_{\bullet }^{(s)}}\nonumber \\&\qquad \Big (\big (E(1-T_{\varDelta })\big )^2 +2E(T_{\varDelta })E\big (T_{\varDelta }(1-T_{\varDelta })\big ) (1-f_{\text{ v }})z_1z_2\nonumber \\&\qquad +\,2E(T_{\varDelta })E\big (T_{\varDelta }(1-T_{\varDelta })\big )f_{\text{ v }}\nonumber \\&\qquad +\,2E(T_{\varDelta })E\big ((1-T_{\varDelta })^2\big )z_1 +E(T_{\varDelta })^2z_1^2\Big )^{\varDelta _{\bullet }^{(s)}}\bigg ]. \end{aligned}$$

Similarly

29 $$\begin{aligned} E(z_1^{\zeta _{2,1}}z_2^{\zeta _{2,2}}z_3^{\zeta _{2,3}})= & {} E\bigg [\Big (E(T_{s})z_3+1-E(T_{s})\Big )^{S_{\bullet }^{(\varDelta )}}\nonumber \\&\Big (\big (E(1-T_{\varDelta })\big )^2 +2E(T_{\varDelta })E\big (T_{\varDelta }(1-T_{\varDelta })\big ) (1-f_{\text{ v }})z_1z_2\nonumber \\&\quad +\,2E(T_{\varDelta })E\big (T_{\varDelta }(1-T_{\varDelta })\big )f_{\text{ v }}\nonumber \\&\quad +\,2E(T_{\varDelta })E\big ((1-T_{\varDelta })^2\big )z_1 +E(T_{\varDelta }s)^2z_1^2\Big )^{\varDelta _{\bullet }^{(\varDelta )} }\bigg ]. \end{aligned}$$

and

30 $$\begin{aligned} E(z_1^{\zeta _{1,1}}z_2^{\zeta _{1,2}}z_3^{\zeta _{1,3}})=f_{\text{ v }}+(1-f_{\text{ v }})E(z_1^{\zeta _{2,1}}z_2^{\zeta _{2,2}}z_3^{\zeta _{2,3}}). \end{aligned}$$

Combining these results yields the probability generating function of the offspring distribution of the three types; $b^{(\text{ v })} (\bar{z})_3$ is given by (28) and $b^{(\text{ v })}(\bar{z})_2$ is given by (29). By replacing $(S^{(s)}_{\bullet }, \varDelta ^{(s)}_{\bullet })$ in the right hand side of (28) by $(S^{(\varDelta )}_{\bullet }, \varDelta ^{(\varDelta )}_{\bullet })$ we obtain $b^{(\text{ v })}(\bar{z})_1$.

Also by replacing $(S^{(s)}_{\bullet }, \varDelta ^{(s)}_{\bullet })$ in the right hand side of (28), but now by $(S,\varDelta )$ we obtain the probability generating function $b_*^{(\text{ v })}(\bar{z})$ of the offspring of the initial case. The expected final size of the epidemic, conditioned on that a major outbreak occurs, is given by

$$\begin{aligned} 1-b^{(\text{ v })}_*(\bar{q}^b). \end{aligned}$$

## Numerical example

Consider for now the special case where $T_{s}=T_{\varDelta }$. With some abuse of notation we denote $T_{s}=T_{\varDelta }$ by *T*, i.e. $T=T_{s}=T_{\varDelta }$ is one-dimensional here. Under very general assumptions, increasing the heterogeneity in infectiousness leads to a decrease in the probability of a major outbreak, the expected final size and $R_0$ (Kuulasmaa 1982; Meester and Trapman 2011; Miller 2008), see also Ball (1985); Kenah and Robins (2007); Miller (2007). In particular, for a fixed (marginal) transmission probability *E*(*T*), the probability of a major outbreak and the expected final size are maximized if $T=E(T)$ with probability 1 and minimized if $P(T=1)=E(T)=1-P(T=0)$. Similarly, for given *E*(*T*), $R_0$ is maximized if $T=E(T)$ with probability 1 and minimized if $P(T=1)=E(T)=1-P(T=0)$.

We illustrate this with the following two examples. In this first example we assume that $T=T_{s}=T_{\varDelta }$. Consider the three degree distributions

1. $$p(2,1)=1$$
2. $$p(4,0)=0.95=1-p(2,1)$$
3. $$p(0,2)=0.95=1-p(2,1)$$.

That is, in all three degree distributions the total degree is 4 with probability 1. In addition, distribution 1 corresponds to a network where every node is member of exactly one triangle. Distribution 2 corresponds to a network where a node is not a member of any triangle with probability 0.95, while with probability 0.05 a node is member of one triangle. Finally, distribution 3 corresponds to a network where a node is a member of two triangles with probability 0.95, while with probability 0.05 a node is member of one triangle.

Furthermore, let *T* have distribution $\text {Beta}(\alpha , \alpha )$ for some $\alpha >0$. That is, *T* has density, $C_{\alpha }x^{\alpha -1}(1-x)^{\alpha -1}$, on the interval (0, 1), where $C_{\alpha }$ is a normalizing constant. Then $E(T)=1/2$ and we can tune the heterogeneity of the infectivity of infected individuals by varying $\alpha$. In particular

$$\begin{aligned} E(T^2)=\frac{1}{2}\left( 1-\frac{1}{2+\alpha ^{-1}}\right) . \end{aligned}$$

**Fig. 8:**
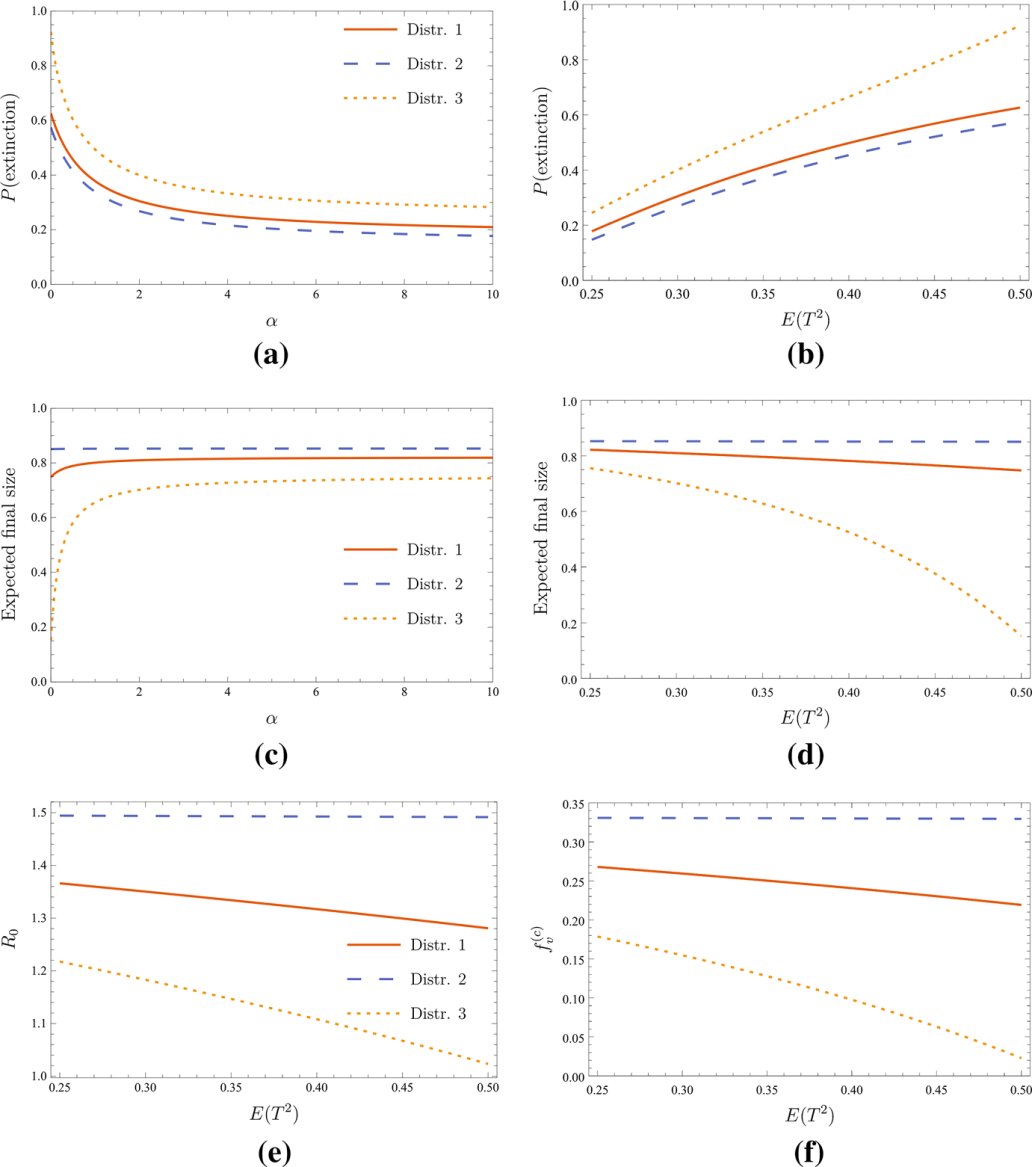
The impact of heterogeneity in infectivity for the three degree distributions. With some abuse of notation we write $T=T_{s}=T_{\varDelta }$. **a** The probability that a major outbreak does not occur as a function of $\alpha$. **b** The probability that a major outbreak does not occur as a function of $E(T^2)$. **c** The expected final size of a major outbreak as a function of $\alpha$. **d** The expected final size of a major outbreak as a function of $E(T^2)$. **e** The basic reproduction number $R_0$ as a function of $E(T^2)$. **f** The critical vaccination coverage $f_v^{(c)}$ as a function of $E(T^2)$

Note that $\alpha =1$ corresponds to $T\sim U(0,1)$, with $\alpha \rightarrow \infty$ corresponding to *T* becoming a point mass at 1 / 2. Here *U*(0, 1) denotes the uniform distribution on (0, 1). Figure 8 shows the probability that a major outbreak does not occur, the expected final size, $R_0$ and the critical vaccination coverage $f_v^{(c)}$ as functions of $\alpha$ or $E(T^2)$.

As can be seen in Fig. 8, ignoring actual heterogeneity of infectivity in this case leads to an overestimation of the probability of a major outbreak (Fig. 8a, b). This effect is particularly evident in the presence of high clustering; the steeper slope of the curve corresponding to distribution 3 (Fig. 8b) and the relatively low probability of a major outbreak when $\alpha$ is small can be explained by the fact that the approximating forward branching process is close to being critical when $\alpha$ is small. Figure 8c, d shows that heterogeneity of infectivity has virtually no impact on the expected final size of a major outbreak and $R_0$ in the near absence of clustering, which is in line with known results for unclustered networks [see, for instance, section 4 in Miller (2008)]. In the presence of clustering, on the other hand, ignoring heterogeneity of infectivity leads to an underestimation of the expected final size and a substantial overestimation of the critical vaccination coverage $f_v^{(c)}$. Note that $R_0$ and $f_v^{(c)}$ depend on the distribution of *T* only through the first and second moment of *T*.

Next, we relax the assumption $T_{s}=T_{\varDelta }$ and investigate the impact of the correlation $\rho$ between $T_{s}$ and $T_{\varDelta }$ on the spread of the disease. To this end, we consider a model where as before $T_{s}$ and $T_{\varDelta }$ both have distribution Beta$(\alpha , \alpha )$ and where the correlation $\rho =\rho (t)$ may be tuned by varying $t\in [-1,1]$. Here $\rho (t)$ is increasing in *t* with $\rho (-1)=-1$ and $\rho (1)=1$. The degree distribution of the underlying graph is given by distribution 1 above.

To construct such a model, let the joint distribution of $T_{s}$ and $T_{\varDelta }$ be as follows. Let $N_1, N_2, N_3$ be three independent standard normal random variables, and assume that the joint distribution of $T_{s}$ and $T_{\varDelta }$ is given by

$$\begin{aligned} T\overset{d}{=}(F_{\alpha }^{-1}(\varPhi (N_s)), F_{\alpha }^{-1}(\varPhi (N_{\varDelta }))), \end{aligned}$$

where $F_{\alpha }$ and $\varPhi$ are the CDF’s of the distributions Beta$(\alpha , \alpha )$ and *N*(0, 1), and $N_s$ and $N_{\varDelta }$ are the standard normal random variables

$$\begin{aligned} N_s= & {} \sqrt{\left| t\right| }N_1+\sqrt{1-\left| t\right| }N_2,\\ N_{\varDelta }= & {} \text {sign}(t)\sqrt{\left| t\right| }N_1 +\sqrt{1-\left| t\right| }N_3. \end{aligned}$$

**Fig. 9:**
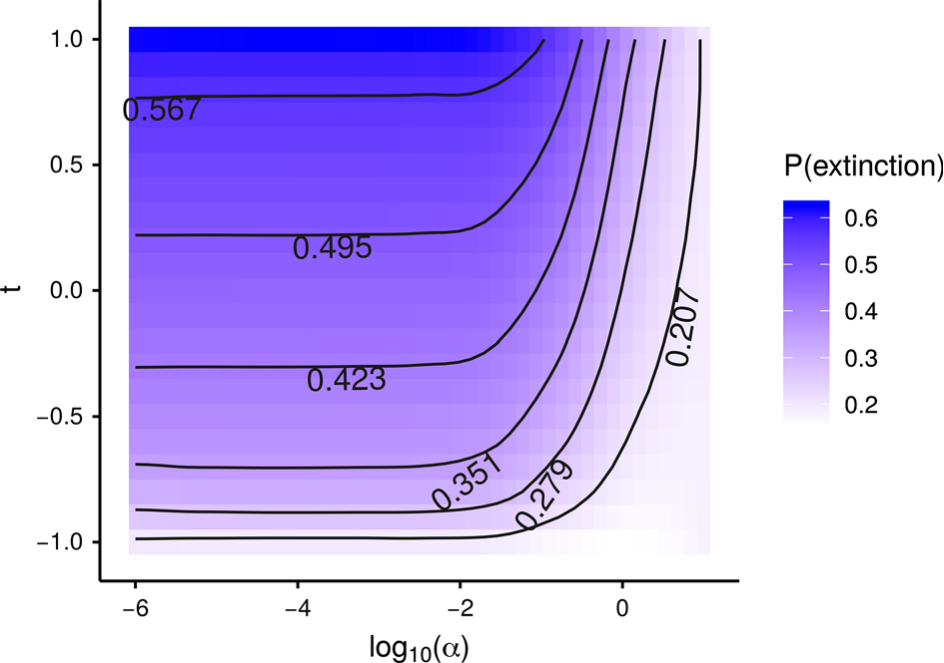
The probability that a major outbreak does not occur as a function of $\alpha$ and *t*

With little effort one can show that $\rho (t)$ is indeed increasing in *t*, that (by the symmetry of the distribution Beta$(\alpha , \alpha )$) $\rho (-1)=-1$ and $\rho (1)=1$, and that $T_{s}$ and $T_{\varDelta }$ are independent for $t=0$. As can be seen in Fig. 9 the probability $1-f^{*}(\bar{q})$ that a major outbreak occurs increases as the correlation $\rho (t)$ decreases. This effect is substantial when heterogeneity in individual infectivity is high (i.e. $\alpha$ is small) but wanes as the heterogeneity decreases (i.e. $\alpha$ increases). This can be explained by the fact that for a fixed value of $\alpha$ the probability that an infectious individual will not transmit the disease to any of its susceptible neighbours decreases as *t* increases, and that this probability is more sensitive to changes in *t* if the heterogeneity in individual infectivity is higher [cf. Kuulasmaa (1982)]. It should be noted that $R_0$ and the critical vaccination coverage $f_v^{(c)}$ do not depend on the correlation $\rho (t)$, which can be seen from the expression for the mean matrix in (9) and (18).

## Discussion

In this paper, we have incorporated clustering in the spread of an infectious disease by allowing for groups of size three with non-overlapping edges. It is, in principle, straightforward to extend the methods used in this paper to larger group sizes. The CMC may, for instance, be generalized to larger group sizes as follows. Let $K=\{k_1,\ldots , k_r\}\subset \mathbb {N}_{\ge 2}$ be the set of possible group sizes. In the matching procedure, each node is equipped with an *r*-dimensional degree in $\mathbb {N}_0^r$. The *i*th component (the $k_i$-degree) of a degree specifies the number of groups of size $k_i$ to which the node in question belongs. Analogously to the construction of a CMC graph, groups are then formed by creating one list for each group size; a node with $k_i$-degree $d_i$ appears precisely $d_i$ times in the list corresponding to groups of size $k_i$. The lists are then shuffled and half-edges of nodes in positions $k+1,\ldots ,k+k_i$ in the $k_i$-list are joined. The structure of a graph so obtained would be characterized by fully connected cliques, and similar to that of a random intersection graph (Ball et al. 2014). One possible approach to investigate epidemics on such graphs would be to approximate the spread of the disease by a multitype Galton Watson process where groups (or cliques) are represented by the particles of the branching process. The types of the approximating branching process would then be vectors in $\mathbb {N}^2$ of the form (*m*, *n*), where *m* represents the size of the clique and *n* represents the number of members of the clique that the primary case of the clique attempts to infect. Another possible approach would be to use an infinite type branching process in the spirit of Ball et al. (2014). We believe that the result would be analogous to the results obtained in Ball et al. (2014).

## Appendix: Proof of proposition 1

Let $\bar{d}=\{(S_i,\varDelta _i)\}_{i\in \mathbb {N}}$ be a given (i.e. non-random) degree sequence that satisfies the following regularity assumptions.

1. $$\frac{\sum _{i=1}^N\mathbb {1}(S_i=k_1, \varDelta _i=k_2) }{N }\rightarrow p(k_1,k_2)$$ for any $$k_1,k_2\in \mathbb {Z}_{\ge 0}$$.
2. $$\frac{\sum _{i=1}^{N}\varDelta _i^2}{N}\rightarrow E(\varDelta ^2) $$ and $$\frac{\sum _{i=1}^{N}S_i^2}{N}\rightarrow E(S^2)$$

where $(S, \varDelta )$ has distribution *p*, which is assumed to satisfy A1–A2 in Sect. 2.1. Let further $\overline{G}=\{G_N\}_{N\in \mathbb {N}}$ be a sequence of graphs generated by the CMC, where the degree sequence of $G_N$ is given by $\bar{d}_N=\{(S_i,\varDelta _i)\}_{i=1}^N$ and denote $D_S^{(N)}=\sum _{i=1}^NS_i$.

Under the assumptions A1–A2 the expected number of self-loops and the expected number of multiple edges are both of order *O*(1) [cf. Van der Hofstad (2016, prop. 7.11)]. Denote by $A_N$ the number of wedges of $G_N$ that are “deleted” when merging multiple edges and erasing self-loops, that is

$$\begin{aligned} A_N=\sum _{i=1}^N{ S_i+2\varDelta _i \atopwithdelims ()2}2-\vert \mathcal {W}^{G_N}_{\wedge }\vert =\sum _{i=1}^N (S_i+2\varDelta _i)(S_i+2\varDelta _i-1)-\vert \mathcal {W}^{G_N}_{\wedge }\vert , \end{aligned}$$

then $$E(A_N)=O(1)$$.

From the definition of $A_N$, we deduce that the total number of ordered triangles of $G_N$ is bounded from below by $\vert \mathcal {W}_{\varDelta }^{G_N}\vert \ge \sum _{i=1}^N 2\varDelta _i-A_N$ and the total number of ordered wedges is bounded from above by

$$\begin{aligned} \vert \mathcal {W}^{G_N}_{\wedge }\vert \le \sum _{i=1}^N{ S_i+2\varDelta _i \atopwithdelims ()2}2=\sum _{i=1}^N (S_i+2\varDelta _i)(S_i+2\varDelta _i-1). \end{aligned}$$

Therefore, by the definition of $C(G_N)$ and the assumptions above

31 $$\begin{aligned} C(G_N)\ge \frac{ \left( \frac{\sum _{i=1}^N 2\varDelta _i}{N} \right) -A_N}{ \left( \frac{\sum _{i=1}^N (S_i+2\varDelta _i) (S_i+2\varDelta _i-1)}{N}\right) }\overset{P}{\rightarrow } \frac{E(2\varDelta )}{E((2\varDelta +S)^2)-E(2\varDelta +S)} \end{aligned}$$

as $$N\rightarrow \infty $$.

This lower bound is tight in the limit as the number of nodes $N\rightarrow \infty$. Indeed, denote by $\mathcal {W}_s^{G_N}$ the set of ordered triangles of $G_N$ that consists solely of single edges, i.e.

$$\begin{aligned} \mathcal {W}_s^{G_N}=\{(u,v,w)\in V_N^3:\ (u,v),(u,w)\text { and} \ (v,w)\ \text {are single edges}\}, \end{aligned}$$

where $V_N$ is the node set of $G_N$. Now, whenever $D_S^{(N)}\ge 6$

32 $$\begin{aligned} E \left( \vert \mathcal {W}_s^{G_N}\vert \right) \le \sum _i\left( { S_i \atopwithdelims ()2} \sum _{j}\frac{S_j}{D_S^{(N)}-2}\left( \sum _{l} \frac{S_l}{D_S^{(N)}-3}\left( \frac{(S_j-1)(S_l-1)}{D_S^{(N)}-5}\right) \right) \right) \end{aligned}$$

where the sums run over the integers $1,\ldots , N$.

Dividing by *N* in (32) and letting *N* approach infinity gives $E(\vert \mathcal {W}_S^{G_N}\vert )/N\rightarrow 0$ as $N\rightarrow \infty$. Thus $\vert \mathcal {W}_S^{G_N}\vert /N{\rightarrow }\ 0$ in probability. Repeating this procedure for triangles formed by a combination of triangle and single edges gives

33 $$\begin{aligned} C(G_N)\overset{P }{\longrightarrow } \frac{E(2\varDelta )}{E((2\varDelta +S)^2)-E(2\varDelta +S)}. \end{aligned}$$

The assertion now follows by bounded convergence and the law of large numbers.

## References

[CR1] Andersson H (1999). Epidemic models and social networks. Math Sci.

[CR2] Ball F (1985). Deterministic and stochastic epidemics with several kinds of susceptibles. Adv Appl Probab.

[CR3] Ball F, Sirl D, Trapman P (2009). Threshold behaviour and final outcome of an epidemic on a random network with household structure. Adv Appl Probab.

[CR4] Ball F, Sirl D, Trapman P (2010). Analysis of a stochastic SIR epidemic on a random network incorporating household structure. Math Biosci.

[CR5] Ball F, Sirl D, Trapman P (2014). Epidemics on random intersection graphs. Ann Appl Probab.

[CR6] Ball F, Pellis L, Trapman P (2016). Reproduction numbers for epidemic models with households and other social structures II: comparisons and implications for vaccination. Math Biosci.

[CR7] Barbour A, Reinert G (2013). Approximating the epidemic curve. Electron J Probab.

[CR8] Bhamidi S, Van der Hofstad R, Komjáthy J (2014). The front of the epidemic spread and first passage percolation. J Appl Probab.

[CR9] Bollobás B (1980). A probabilistic proof of an asymptotic formula for the number of labelled regular graphs. Eur J Comb.

[CR10] Bollobás B, Janson S, Riordan O (2011). Sparse random graphs with clustering. Random Struct Algorithms.

[CR11] Britton T (2010). Stochastic epidemic models: a survey. Math Biosci.

[CR12] Britton T, Janson S, Martin-Löf A (2007). Graphs with specified degree distributions, simple epidemics, and local vaccination strategies. Adv Appl Probab.

[CR13] Britton T, Deijfen M, Lagerås AN, Lindholm M (2008). Epidemics on random graphs with tunable clustering. J Appl Probab.

[CR14] Coupechoux E, Lelarge M (2015). Contagions in random networks with overlapping communities. Adv Appl Probab.

[CR15] Diekmann O, Heesterbeek J, Metz JAJ (1990). On the definition and the computation of the basic reproduction ratio $$R_0$$ in models for infectious diseases in heterogeneous populations. J Math Biol.

[CR16] Diekmann O, Heesterbeek H, Britton T (2013) Mathematical tools for understanding infectious disease dynamics. Princeton University Press, Princeton. http://www.jstor.org/stable/j.cttq9530. Accessed 22 Jan 2018

[CR17] Erdős P, Rényi A (1959). On random graphs I. Publ Math Debrecen.

[CR18] Goldstein E, Paur K, Fraser C, Kenah E, Wallinga J, Lipsitch M (2009). Reproductive numbers, epidemic spread and control in a community of households. Math Biosci.

[CR19] Janson S, Luczak M, Windridge P (2014). Law of large numbers for the SIR epidemic on a random graph with given degrees. Random Struct Algorithms.

[CR20] Karoński M, Scheinerman E, Singer-cohen K (1999). On random intersection graphs: the subgraph problem. Comb Probab Comput.

[CR21] Kenah E, Miller J (2011) Epidemic percolation networks, epidemic outcomes, and interventions. Interdiscip Perspect Infect Dis. 10.1155/2011/543520 PMC3062991 21437002

[CR22] Kenah E, Robins JM (2007). Second look at the spread of epidemics on networks. Phys Rev E.

[CR23] Kiss I, Miller J, Simon P (2017) Mathematics of epidemics on networks: from exact to approximate models. Springer, Berlin

[CR24] Kuulasmaa K (1982). The spatial general epidemic and locally dependent random graphs. J Appl Probab.

[CR25] Ludwig D (1975). Final size distribution for epidemics. Math Biosci.

[CR26] Meester R, Trapman P (2011). Bounding the size and probability of epidemics on networks. Adv Appl Probab.

[CR27] Meyers L (2007). Contact network epidemiology: bond percolation applied to infectious disease prediction and control. Bull Am Math Soc.

[CR28] Miller J (2007). Epidemic size and probability in populations with heterogeneous infectivity and susceptibility. Phys Rev E.

[CR29] Miller J (2008). Bounding the size and probability of epidemics on networks. J Appl Probab.

[CR30] Miller J (2009). Percolation and epidemics in random clustered networks. Phys Rev E.

[CR31] Molloy M, Reed B (1995). A critical point for random graphs with a given degree sequence. Random Struct Algorithms.

[CR32] Molloy M, Reed B (1998). The size of the giant component of a random graph with a given degree sequence. Comb Probab Comput.

[CR33] Newman M (2002). Spread of epidemic disease on networks. Phys Rev E.

[CR34] Newman M (2003). Properties of highly clustered networks. Phys Rev E.

[CR35] Newman M (2009). Random graphs with clustering. Phys Rev Lett.

[CR36] Newman M, Watts D, Strogatz S (2002). Random graph models of social networks. Proc Natl Acad Sci USA.

[CR37] Pellis L, Ferguson NM, Fraser C (2008). The relationship between real-time and discrete-generation models of epidemic spread. Math Biosci.

[CR38] Pellis L, Ball F, Trapman P (2012). Reproduction numbers for epidemic models with households and other social structures. I. Definition and calculation of $$R_0$$. Math Biosci.

[CR39] Trapman P (2007). On analytical approaches to epidemics on networks. Theor Popul Biol.

[CR40] Van der Hofstad R (2016). Random graphs and complex networks.

[CR41] Varga RS (2009) Matrix iterative analysis. Springer, Berlin. 10.1007/978-3-642-05156-2

[CR42] Volz E, Miller J, Galvani A, Meyers L (2011). Effects of heterogeneous and clustered contact patterns on infectious disease dynamics. PLoS Comput Biol.

